# Genetic Variation in Virulence among Chalkbrood Strains Infecting Honeybees

**DOI:** 10.1371/journal.pone.0025035

**Published:** 2011-09-22

**Authors:** Svjetlana Vojvodic, Annette B. Jensen, Bo Markussen, Jørgen Eilenberg, Jacobus J. Boomsma

**Affiliations:** 1 Centre for Social Evolution, Faculty of Life Sciences, Department of Agriculture and Ecology, University of Copenhagen, Copenhagen, Denmark; 2 Department of Natural Sciences and Environment, Faculty of Life Sciences, University of Copenhagen, Copenhagen, Denmark; 3 Centre for Social Evolution, Department of Biology, University of Copenhagen, Copenhagen, Denmark; College of Medicine, Hallym University, Republic of Korea

## Abstract

*Ascosphaera apis* causes chalkbrood in honeybees, a chronic disease that reduces the number of viable offspring in the nest. Although lethal for larvae, the disease normally has relatively low virulence at the colony level. A recent study showed that there is genetic variation for host susceptibility, but whether *Ascosphaera apis* strains differ in virulence is unknown. We exploited a recently modified *in vitro* rearing technique to infect honeybee larvae from three colonies with naturally mated queens under strictly controlled laboratory conditions, using four strains from two distinct *A. apis* clades. We found that both strain and colony of larval origin affected mortality rates. The strains from one clade caused 12–14% mortality while those from the other clade induced 71–92% mortality. Larvae from one colony showed significantly higher susceptibility to chalkbrood infection than larvae from the other two colonies, confirming the existence of genetic variation in susceptibility across colonies. Our results are consistent with antagonistic coevolution between a specialized fungal pathogen and its host, and suggest that beekeeping industries would benefit from more systematic monitoring of this chronic stress factor of their colonies.

## Introduction

Hosts and parasites are often intertwined in arms races, but antagonistic co-evolution can only take place if the necessary genetic variation in host susceptibility and parasite virulence is available for selection. Such conditions have been extensively modelled e.g. [1] and shown to apply in empirical studies e.g. [2]. Colonies of social insects are peculiar as hosts because individual immune defences are supplemented by collective behavioural defences such as social fever and targeted hygienic behaviour [3]–[5]. Immune defences of social insect colonies have further been shown to benefit from genetic heterogeneity owing to multiple insemination of queens in honeybees [6], ants [7], and bumblebees [8].

Honeybee colonies face considerable risks of reduced productivity and colony failure due to parasites [3]–[6]. However, most studies have focused on high virulence diseases such as American foulbrood, caused by *Paenibacillus larvae* bacteria [9], whereas low virulence diseases have been relatively neglected in spite of them being rather common [3]. These less virulent parasites are relevant as stress factors that may contribute to colony collapse disorder [10], and at the same time they provide unique opportunities for studying co-evolutionary dynamics. Chalkbrood is one such low virulence disease [11], [12], caused by the fungus *Ascosphaera apis*, killing honeybees larvae after spore ingestion. Whether chalkbrood strains differ in virulence similarly to other honeybee parasites such as American foulbrood is unknown [9]. The objective of our study was to address this question by exploiting both the availability of an extensive chalkbrood strain collection and modified *in vitro* rearing technique for honeybee larvae [12]. We evaluate the consequences of our findings both for understanding co-evolutionary dynamics of honeybee diseases and for practical beekeeping.

## Materials and Methods

### Pathogen isolation

Twenty Danish *A. apis* strains were isolated from honeybees mummies collected by Danish bee keepers. Infected larvae were surface sterilized in 10% sodium hypochlorite for 10 min followed by 2 min. water washing [13]. Rinsed larvae were cut into three pieces and placed on Sabouraud Dextrose Agar (SDA) growth medium at 34°C. After several days the *A. apis* mycelia were observed growing on the agar plates. Single hyphal tips were isolated with a sterile scalpel using a dissecting microscope. Each hyphal tip was placed on a new Petri dish with SDA growth medium, incubated at 34°C until growth was observed and stored at 25°C for 2 weeks. For long term storage mycelia were placed in 20% glycerol at −80°C [14].

### DNA extraction, PCR amplification and sequencing

Genomic DNA from *A. apis* isolates was extracted from lyophilized hypae using the DNeasy® Plant Mini Kit (Qiagen) and DNA extracts were diluted 1∶10 in sterile MilliQ water prior to polymerase chain (PCR) reaction. PCR amplification was conducted for a variable part of the EF1α and two intergenic regions located on scaffolds 300 and 1635 of the assembled *A. apis* genome sequence [15]. Samples for PCR amplifications consisted of 1 U Phusion® High-Fidelity DNA Polymerase (New England Biolabs, Inc.) with appropriate buffer (HF buffer (1.5 mM MgCL2), 0.2 µm dNTPs, 1 µm of each forward and reverse primer, in a final reaction volume 50 µL. All reactions were carried out on a T1 Thermocycler using a touchdown approach with cycling conditions consisting of: 30 s denaturation at 98°C; 10 cycles at 98°C for 30 s; 70–60 cycles (decrease of 1°C per cycle) for 30 s and 72°C for 30 s; 30 cycles of 98°C for 30 s, 60°C for 30 s, 72°C for 30 s, with a final 10 min extension at 72°C. PCR products were electrophoretically separated on 1.5% agarose gels, visualised with EZ vision One® (Amresco), cleaned with an illustra GFX™ PCR DNA and Gel Band Purification Kit (GE-Healthcare) and sent to Eurofins MWG Operon AG, Ebersberg, Germany for sequencing with both forward and reverse primers.

### DNA sequence analyses

Sequences were edited and aligned manually using BioEdit [16] and sequence analysis of alignments were conducted in MEGA version 4 [17] on a dataset combining all three loci (EF1a, scaffold 300 and scaffold 1634) using the Neighbor-Joining method with a pairwise deletion option. Evolutionary distances between strains were computed using the Jukes-Cantor method and branch support values were assessed by bootstrapping of 1000 replicate datasets. Further information on the strain collection can be found in Table S1.

### Maintenance of *Ascosphaera apis* cultures and inoculum preparation

*Ascosphaera apis* is a heterothallic fungus, meaning that production of spores only occurs when the hyphae of both mating types are in contact. Therefore each isolated strain had to be mated with the characterized strain ARSEF 7405 or ARSEF 7406 (USDA-ARS Collection of Entomopathogenic Fungal Cultures in Ithaca, New York, USA). Once the strains were designated a mating type, they were paired and placed on a Petri dish. We chose 4 pairs of strains designated A (KVL06-150, KVL06-158), D (KVL06-182, KVL08-41), F (KVL06-123, KVL06-132), and G (KVL07-087, KVL07-104). The two paired strains A and D came from one phylogenetic clade and the paired strains F and G from another clade (see below).

In order to obtain fresh spores 3 weeks prior to the experiment, isolated strains from each phylogenetic clade were paired. The produced spores were removed from the plates with a small sterile spatula and placed into a sterile glass grinder with 20 µl of sterile deionized water. Following the grinding, 50 µl of sterile deionized water was added to the spore suspension. Large particles in the suspension were allowed to settle for 20 min, and a sample of approximately 50 µl was taken from the middle of the suspension. Spore concentration in the resulting suspension was determined with a hemocytometer (Tiefe Depth Profondeur, Marienfeld, Germany).

### Spore viability

Spore viability for *A. apis* was tested following the protocol of James and Buckner [18] with a few modifications. A spore suspension (150 µl) of a concentration of 2×10^7^ spores per ml was mixed with 150 µl GLEN, a liquid medium suitable for germination and *in vitro* growth of insect pathogenic fungi [19]. Droplets of 10 µl of mixture were placed on three spots of a sterile Teflon coated slide, which was deposited in a sterile Petri dish lined with wet filter paper. Each Petri dish was subsequently placed in an airtight container flushed with CO_2_. The containers were incubated for 24 hours at 34°C, after which the Teflon coated slide received a cover-slip and the spore germination percentage was determined using differential interference contrast microscopy at 400x magnification. One hundred spores were evaluated for enlargement or germ tube formation in three different randomly chosen fields of view. Overall, the spore germination rates ranged from 10 to 20%.

### Host maintenance and *in vitro* rearing

Honeybee (*A. mellifera*) larvae were obtained from an apiary located at the University of Copenhagen. Colonies were checked regularly and were free of any noticeable brood and adult bee diseases. For each experiment larvae were transferred from the three hives and reared *in vitro* following the protocol of Aupinel et al. [20] and Vojvodic et al. [12] with a few modifications. Larval age was estimated by size [21] and larvae that were 24 h old (+/− 6 hours) were taken from the combs using a Swiss grafting tool (Swienty, Sønderborg, Denmark). After removal from the comb each larva was placed into an individual cell of a 48-well tissue culture plate with 10 µl of larval diet. The larval diet consisted of 50% of Chinese fresh frozen royal jelly (v/v) (Sonnentracht Imkerei GmbH, Bremen, Germany), 6% D-glucose (w/v), 6% D-fructose (w/v), 1% (w/v) yeast extract and sterile deionised water. The diet was mixed and frozen in smaller aliquots and was pre-heated to 34°C before being used for feeding. The larvae were fed once a day with 20 µl on the first three days, and 40 µl on day four. The tissue culture plates with the larvae were stored in a humid chamber and incubated at 34°C in constant darkness. Wells were gently cleaned with cotton wool in case larvae started to defecate.

### Host inoculation

Two days before the experiment larvae were removed from each of three hives with unrelated queens and reared *in vitro* as described above. After a 48 h acclimatization period, 30 healthy larvae were fed 5 µl of a designated spore suspension of one of the pathogen strains (A, D, F, or G) using 5×10^5^ spores/ml and distilled water (in the case of the control). In total, 360 honeybee larvae from three hives were exposed to one of the 4 genetically distinct *A. apis* strains and 90 larvae were treated with distilled water as a control. To avoid any temporal and environmental differences the experiment was set up at one time period, limiting the number of colonies as well as individual bees that could have been handled simultaneously. Within one day, the larvae had ingested all food, including the spores. The possibility of spores present after day one, and the risk of later infections were thus minimized. The experimental larvae were kept in a humid chamber at a constant temperature of 34°C for 7 days. The number of diseased, surviving, and infected larvae were examined microscopically and recorded daily. Infected host larvae were identified by ceased respiration, loss of body elasticity, or a change to gray or brown colors, and fungal hyphae on the cuticle. Larvae that died without any visual presence of fungal hyphae were re-examined the following day. If the pathogen was observed protruding through the host cuticle, these larvae were considered dead from the pathogen on their initial day of death. If the pathogen was not visually present on dead larvae, they were recorded as dead from natural causes.

### Statistical analysis - Survivorship analysis

Statistical analyses were done using the proportional hazard model (also known as Cox regression) analyzing the event times at the day of death, and censoring times at the termination of the study on day 8 [22]. In this model the instantaneous hazards of dying from the infection were described as functions of time, colony, and strain. The proportional hazard null model was that the instantaneous hazards were proportional across hives and strains when considered as functions of time. Prior to formal hypothesis testing, this assumption was validated using the methods proposed in Lin et al. [23]. Model reduction was done using likelihood ratio tests starting from the initial model including the main effects of hive and strain together with their interaction. Post-hoc comparisons were done using Wald tests. Since the larvae were observed only once a day, several larvae were sometimes observed to have died at the same time. Such observational ties were analyzed by averaging over the event times and under the assumption that the censored times have taken place after the event times. All computations were done using SAS V9.2. Bonferonni correction for multiple testing was used to adjust the reported p-values for post-hoc comparisons.

## Results

**Figure 1:**
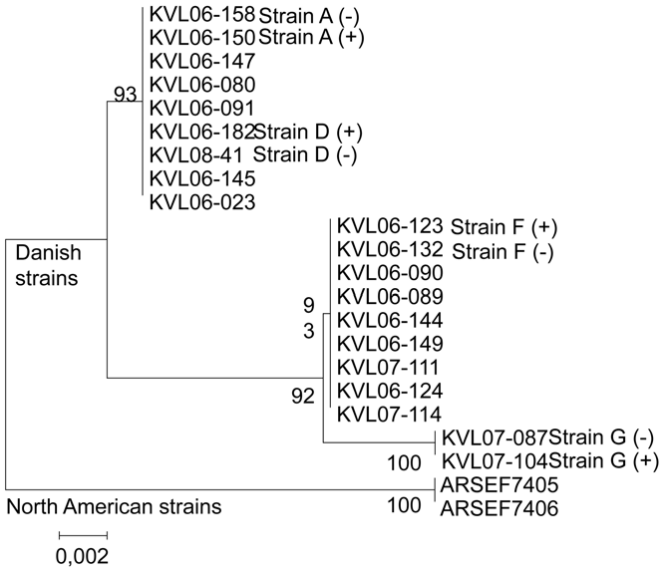
Phylogenetic relationships among the *Ascosphaera apis* strains A, D, F, and G used in the larval exposure experiments. Positive (+) and negative (–) symbols indicate different mating types used to obtain sporulating clade-specific heterokaryons.

DNA sequences of parts of the EF1α gene and the two intergenic regions were obtained for a total of 2015 nucleotide positions at 71 variable sites and could be included in the alignment that produced the combined dataset. The twenty Danish isolates were grouped into clusters with two reference isolates (from the USDA-ARS Collection of Entomopathogenic Fungal Cultures) as outgroup (Figure 1). Each clade was supported by bootstrap values of ≥92%.

The asymptotic chi-square distribution of twice the log likelihood ratio showed that the interaction between hives and strains was not significant (LR = 5.717; df = 5; p = 0.4556), but that the factors “strain” (LR = 15.941; df = 2; p =  0.0003) and “hive” (LR = 47.285; df = 3; p<0.0001) were both highly significant predictors of infection-induced mortality. Visible signs of infection were recorded as early as Day 3 for strain F, and Day 4 for the other strains (Figure 1). Strains A and D caused relatively low host mortality of 12 and 14% on the last day of the experiment, respectively, whereas strains F and G induced larval mortality of 92 and 71% on that day, respectively. Detailed information on larval survival are included in Table S2.

**Figure 2:**
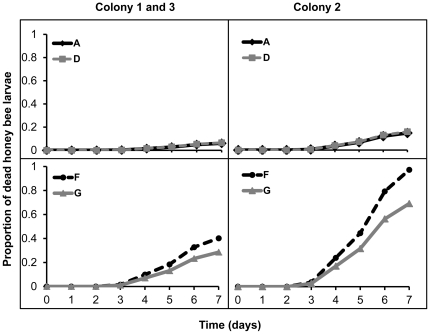
Cumulative proportions of honeybee larvae dead from chalkbrood disease after exposure to the four *Ascosphaera apis* strains A, D, F and G. Larvae from Colony 2 were significantly more susceptible than larvae from Colonies 1 and 3 (pooled after statistical analysis). Strains F and G were significantly more virulent than Strains A and D, but the respective virulence of Stain A and D and Strain F and G were not significantly different from each other.

Pairwise comparisons (Figure 2) showed that strains A and D were not significantly different in virulence (Wald  = 0.02; p = 5.337), and that strains F and G were also not statistically different (Wald  = 1.89; p = 1.0134) (Table 1). However, strain A differed significantly from strains F (Wald  = 20.83, p<0.0006) and G (Wald  = 13.57, p = 0.0012) and strain D showed a similarly reduced virulence relative to strains F (Wald  = 21.52, p<0.0006) and G (Wald  = 13.74, p = 0.0012). Furthermore, colonies 1 and 3 were not significantly different in their susceptibility to *A. apis* strains (Wald =  8.1108; p = 1.6494), whereas colony 2 was significantly more susceptible (Figure 2).

**Table 1:** Pairwise comparisons evaluating the virulence of *Ascosphaera apis* strains.

Variable		Hazard ratio	95% CI	χ^2^	Corrected P
**Strains**					
	Strain D vs Strain A	1.08	0.39–3.07	0.0193	5.337
	Strain G vs Strain A	4.71	2.19–11.66	13.5700	**0.0012**
	Strain F vs Strain A	6.62	3.13–16.25	20.8383	**< 0.0006**
	Strain F vs Strain D	6.16	3.01–14.28	21.5207	**< 0.0006**
	Strain G vs Strain D	4.38	2.11–10.25	13.7430	**0.0012**
	Strain F vs Strain G	1.41	0.86–2.27	1.8926	1.0134
**Colony**					
	Colony 3 vs Colony 1	1.22	1.22–0.64	0.3576	1.6494
	Colony 2 vs Colony 1	2.67	2.67–1.58	12.5807	**0.0012**
	Colony 3 vs Colony2	0.46	0.26–0.77	8.1108	**0.0132**

Confidence intervals are 95% Hazard Ratio Profile Likelihood Limits. Significant differences are given in bold-faced print. Bonferonni correction was used to adjust the p-values.

## Discussion

We found significant variation in virulence between four Danish chalkbrood strains from two distinct clades and evidence for variation in susceptibility between the three host hives. As all three colonies were of the same size and came from the same apiary, we infer that these susceptibility differences likely reflect genetic rather than environmental variation, as was documented earlier by Tarpy [24]. Our results are therefore consistent with the presence of relevant genetic variation, for both host and parasite, as required for antagonistic host-parasite co-evolution.

Evolutionary studies e.g. [25], [26] tend to predict intermediate virulence levels, with exact levels for any system depending on transmission mode and the frequency of multiple infection. While this has been shown to some degree in bumblebees [8], it has also become clear that these inferences may not necessarily apply for all social insect hosts when prophylactic social behaviours interact with disease defences at the level of individual larvae [27]. Honeybees are known to be able to detect chalkbrood diseased larvae and remove them from their cells [5]. This might imply that more virulent strains produce infected host larvae that can be more efficiently discarded by workers before spore transmission. Future work should therefore establish if highly virulent strains might bear a higher cost due of premature detection, so that behavioural responses may affect the transmission and effective virulence of strains and thus help to maintain genetic variation for virulence.

A close relative of honeybee chalkbrood, *Ascosphaera aggregata*, causes chalkbrood in solitary *Megachile rotundata* bees [28] indicating that chalkbrood fungi and bees have a long co-evolutionary history. However, while leafcutter bees have small annual nests containing a singly mated female and her offspring, honeybees have large, perennial and highly complex societies, headed by a multiply mated queen. Polyandry causes higher genetic variation that has been shown to enhance overall colony performance [29] and to reduce parasite prevalence [3] for both chalkbrood [24] and American foulbrood [6]. Invernizzi et al. [30] further showed that there is significant variation between patrilines for chalkbrood resistance when larvae are infected with spores from dead larvae in the field, i.e. with inoculates that potentially harbour numerous strains.

Invernizzi et al. [30] did not control for parasite genotype but investigated variation in resistance between patrilines within honeybee colonies, indicating genetic variation for larval resistance. Alternatively, we controlled for parasite genotype, focusing on variation in virulence between parasite strains of known genotype together with between-colony variation in host resistance. Our results indicate genetic variation for parasite virulence, while the between-colony variation in host resistance that we observed is suggestive of host genetic variation for resistance. Future studies that seek to understand host –parasite coevolution should consider strictly controlling all aspects of both host and parasite genotypes. In the case of social insects, this also includes social immunity, where social interactions with nestmates can provide heritable social lines of defence to combat diseases beyond normal innate immune responses [31].

Given the high commercial value of honeybees as pollinators and honey producers, it is surprising that so little work has been done on genetic variation in susceptibility and virulence of common chronic diseases such as chalkbrood. In times of significant but poorly understood declines of honeybee stocks worldwide, a better understanding of the stress factors due to relatively mild diseases should be a high priority. Although evolutionary trade-offs may prevent the evolution of higher resistance to chalkbrood via natural selection, our study shows that relevant genetic variation in virulence could potentially be used in honeybee artificial selection programs.

## Supporting Information

Table S1 Reference sequence information for *Ascosphaera apis* strains.
(DOC)

Table S2 The number of honeybee larvae dead from *Ascosphaera apis* infections with strains A, D, F and G. All treatment and control combinations consisted of 90 larvae, whose mortality was censured during seven consecutive days. The columns towards the right give the total numbers of surviving larvae throughout the observation period and the numbers of dead larvae due to natural and disease causes. See Fig. 2 for cumulative proportions.
(DOCX)
