# Supplementary material for: Genetic Variation in Virulence among Chalkbrood Strains Infecting Honeybees
Source: PLoS One. 2011 Sep 22;6(9):e25035. doi: 10.1371/journal.pone.0025035 (PMC3178585; doi:10.1371/journal.pone.0025035)
Supplement: Table S1 — Reference sequence information for Ascosphaera apis strains. (DOC) [file pone.0025035.s001.doc]

| ***Isolate no.*** | ***Origin*** | ***EF1α*** | ***Scaffolds 300*** | ***Scaffolds 1635*** |
| --- | --- | --- | --- | --- |
| KVL06-023 | Denmark, 3300 Frederiksværk | HQ905489 | HQ905511 | HQ905533 |
| KVL06-080 | Denmark, 4100 Ringsted | HQ905490 | HQ905512 | HQ905534 |
| KVL06-089 | Denmark, 5856 Rysling | HQ905491 | HQ905513 | HQ905535 |
| KVL06-090 | Denmark, 8963 Auning | HQ905492 | HQ905514 | HQ905536 |
| KVL06-091 | Denmark, 4100 Ringsted | HQ905493 | HQ905515 | HQ905537 |
| KVL06-123 | Denmark, 4920 Søllested | HQ905494 | HQ905516 | HQ905538 |
| KVL06-124 | Denmark, 4920 Søllested | HQ905495 | HQ905517 | HQ905539 |
| KVL06-132 | Denmark, 7700 Klitmøller | HQ905496 | HQ905518 | HQ905540 |
| KVL06-144 | Denmark, 8700 Horsens | HQ905497 | HQ905519 | HQ905541 |
| KVL06-145 | Denmark, 3390 Hundested | HQ905498 | HQ905520 | HQ905542 |
| KVL06-147 | Denmark, 2100 København | HQ905499 | HQ905521 | HQ905543 |
| KVL06-149 | Denmark, 1885 Frederiksberg | HQ905500 | HQ905522 | HQ905544 |
| KVL06-150 | Denmark, 3390 Hundested | HQ905501 | HQ905523 | HQ905545 |
| KVL06-158 | Denmark, 3390 Hundested | HQ905502 | HQ905524 | HQ905546 |
| KVL06-182 | Denmark, 4653 Karise | HQ905503 | HQ905525 | HQ905547 |
| KVL07-087 | Denmark, 8963 Auning | HQ905504 | HQ905526 | HQ905548 |
| KVL07-104 | Denmark, 8070 Hadsten | HQ905505 | HQ905527 | HQ905549 |
| KVL07-111 | Denmark, 8700 Horsens | HQ905506 | HQ905528 | HQ905550 |
| KVL07-114 | Denmark, 2800 Lyngby | HQ905507 | HQ905529 | HQ905551 |
| KVL08-041 | Denmark, 3700 Rønne | HQ905508 | HQ905530 | HQ905552 |
| ARSEF7405 | USA, Weslaco, Texas | HQ905487 | HQ905509 | HQ905531 |
| ARSEF7406 | USA, Weslaco, Texas | HQ905488 | HQ905510 | HQ905532 |
