# Supplementary material for: Genetic Variation in Virulence among Chalkbrood Strains Infecting Honeybees
Source: PLoS One. 2011 Sep 22;6(9):e25035. doi: 10.1371/journal.pone.0025035 (PMC3178585; doi:10.1371/journal.pone.0025035)
Supplement: Table S2 — The number of honeybee larvae dead from Ascosphaera apis infections with strains A, D, F and G. All treatment and control combinations consisted of 90 larvae, whose mortality was censured during seven consecutive days. The columns towards the right give the total numbers of surviving larvae throughout the observation period and the numbers of dead larvae due to natural and disease causes. See Fig. 2 for cumulative proportions. (DOCX) [file pone.0025035.s002.docx]

| **Treatments** | **Number of dead larvae on each day** | | | | | | | **Total** | |
| --- | --- | --- | --- | --- | --- | --- | --- | --- | --- |
|  | **1** | **2** | **3** | **4** | **5** | **6** | **7** | **Surviving** | **Dead from *A. apis*** |
| **Control** | 0 | 0 | 0 | 0 | 0 | 0 | 0 |  |  |
| **Natural mortality** | 0 | 0 | 1 | 4 | 2 | 4 | 1 | 78 | 0 |
| **Strain A** | 0 | 0 | 0 | 1 | 3 | 3 | 0 | 57 | 7 |
| **Natural mortality** | 3 | 1 | 5 | 4 | 2 | 4 | 7 |  |  |
| **Strain D** | 0 | 0 | 0 | 2 | 1 | 3 | 2 | 58 | 8 |
| **Natural mortality** | 0 | 0 | 3 | 6 | 3 | 4 | 8 |  |  |
| **Strain F** | 0 | 0 | 4 | 11 | 6 | 12 | 3 | 39 | 36 |
| **Natural mortality** | 2 | 0 | 2 | 4 | 1 | 5 | 1 |  |  |
| **Strain G** | 0 | 0 | 0 | 7 | 8 | 9 | 6 | 42 | 30 |
| **Natural mortality** | 1 | 2 | 1 | 4 | 2 | 1 | 7 |  |  |
| **Total** |  |  |  |  |  |  |  |  |  |
| ***A. apis*** | 0 | 0 | 4 | 21 | 18 | 27 | 11 | 24 | 81 |
| **Natural mortality** | 6 | 3 | 12 | 22 | 10 | 18 | 24 | 274 | 81 |
